# Quantitative differences in volumetric calculations for radiation dosimetry in segmental Y90 treatment planning using hybrid angiography-CT compared with anatomic segmentation

**DOI:** 10.1093/bjr/tqad056

**Published:** 2024-01-04

**Authors:** Daniel H Kwak, Alex Lionberg, Mikin Patel, Karan Nijhawan, Spencer Martens, Qian Yu, David Cao, Salma Youssef, Osman Ahmed

**Affiliations:** Department of Radiology, Section of Interventional Radiology, The University of Chicago Medical Center, Chicago, IL 60637, United States; Department of Radiology, Section of Interventional Radiology, The University of Chicago Medical Center, Chicago, IL 60637, United States; Department of Radiology, Section of Interventional Radiology, The University of Chicago Medical Center, Chicago, IL 60637, United States; Department of Radiology, Section of Interventional Radiology, The University of Chicago Medical Center, Chicago, IL 60637, United States; Department of Radiology, Section of Interventional Radiology, The University of Chicago Medical Center, Chicago, IL 60637, United States; Department of Radiology, Section of Interventional Radiology, The University of Chicago Medical Center, Chicago, IL 60637, United States; The University of Chicago Pritzker School of Medicine, Chicago, IL 60637, United States; University College Dublin School of Medicine, Dublin 4, Ireland; Department of Radiology, Section of Interventional Radiology, The University of Chicago Medical Center, Chicago, IL 60637, United States

**Keywords:** selective internal radiation therapy, transarterial radioembolization, yttrium-90 dosimetry, Angio-CT, interventional oncology

## Abstract

**Objective:**

To compare treatment volumes reconstructed from hybrid Angio-CT catheter-directed infusion imaging and Couinaud anatomic model as well as the implied differences in Y-90 radiation dosimetry.

**Methods:**

Patients who underwent transarterial radioembolization (TARE) using Y-90 glass microspheres with pretreatment CT or MRI imaging as well as intraprocedural angiography-CT (Angio-CT) were analysed. Treatment volumes were delineated using both tumoural angiosomes (derived from Angio-CT) and Couinaud anatomic landmarks. Segmental and lobar treatment volumes were calculated via semi-automated contouring software. Volume and dose differences were compared by the two-tailed Student *t* test or Wilcoxon signed-rank test. Factors affecting volume and dose differences were assessed via simple and/or multiple variable linear regression analysis.

**Results:**

From September 2018 to March 2021, 44 patients underwent 45 lobar treatments and 38 patients received 56 segmental treatments. All target liver lobes and all tumours were completely included within the field-of-view by Angio-CT. Tumour sizes ranged between 1.1 and 19.5 cm in diameter. Segmental volumes and treatment doses were significantly different between the Couinaud and Angio-CT volumetry methods (316 vs 404 mL, *P *<* *.0001 and 253 vs 212 Gy, *P *<* *.01, respectively). Watershed tumours were significantly correlated with underestimated volumes by the Couinaud anatomic model (*P *<* *.001). There was a significant linear relationship between tumour diameter and percent volume difference (*R*^2^ = 0.44, *P *<* *.0001). The Couinaud model overestimated volumes for large tumours that exhibited central hypovascularity/necrosis and for superselected peripheral tumours.

**Conclusions:**

Angio-CT may confer advantages over the Couinaud anatomic model and enable more accurate, personalized dosimetry for TARE.

**Advances in knowledge:**

Angio-CT may confer advantages over traditional cross-sectional and cone-beam CT imaging for selective internal radiation therapy planning.

## Introduction

There is growing evidence that a personalized dosimetry approach for selective internal radiation therapy (SIRT), also known as yttrium-90 (Y-90) transarterial radioembolization (TARE), is superior to standard dosimetry.^1–3^ Accurate assessment of treatment area volume is critical to personalized dosimetry, and under- or overdosing can result in unfavourable treatment outcomes^1–3^ (objective response, overall response rates, and overall survival) or TARE-related complications (post-radioembolization syndrome,^4^ radiation-induced liver disease,^5^ and radiation pneumonitis,^5–7^ respectively). Treatment area volumes derived from the Couinaud anatomic model rely on portal and venous anatomic landmarks; however, they may not accurately represent the arterially perfused treatment area volume. Volumetric analyses based on perfused treatment area volume of liver tissue from the point of radiomicrosphere infusion (“tumoural angiosome”) derived from intraprocedural cone-beam CT (CBCT) imaging data have demonstrated advantages for both Y-90 embedded glass^8^ and resin^9^ microspheres.

Prior studies have demonstrated significant variability and deviation between dosimetry derived from CBCT volumetric data and treatment area volume from the Couinaud anatomic model.^8^^,^^9^ Factors accounting for these differences included microcatheter positioning (proximal vs distal), variant arterial anatomy, and tumour location (on or near segmental watersheds).^8^ However, these studies are limited by the use of qualitative and descriptive analyses as well as lack of statistical rigour to establish the significance of these variables. Angiography-CT (Angio-CT) integrates fluoroscopic angiography with traditional fan-beam CT in a single procedural suite. Whilst its utility in interventional oncology is well established, there is a paucity of published data regarding its use for TARE specifically.^10^ Angio-CT confers several benefits over CBCT, including untruncated field-of-view, superior contrast-to-noise ratio, improved contrast timing, reduced patient radiation exposure, and lower susceptibility to artefacts (respiratory motion, beam hardening, and ring).^11–18^ These advantages may thus allow for more reliable assessment of tumoural angiosomes regardless of liver size or patient cooperation as prior studies utilizing CBCT excluded up to 27% of patients due to limitations in field-of-view.^8^ The objective of this study was to analyse discrepancies between treatment volumes reconstructed from Angio-CT catheter-directed infusion image data and those derived from the Couinaud anatomic model.

## Methods

### Study design and patient selection

**Figure 1.**
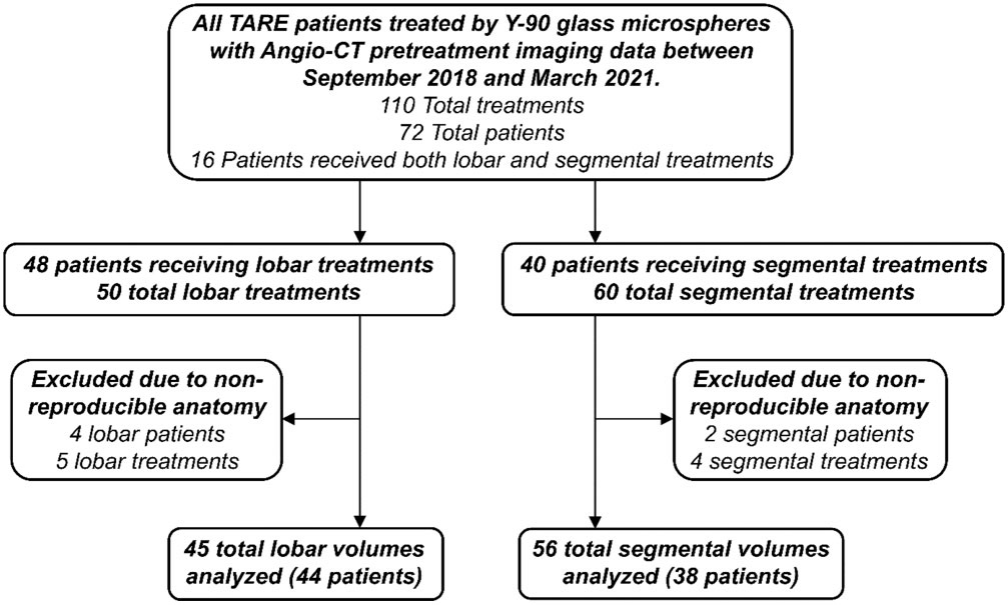
Study design and analysis of lobar and segmental treatment volumes.

An IRB-approved, retrospective analysis was performed on all patients who underwent TARE via Y-90 embedded glass microspheres at a single academic institution between September 2018 and March 2021 (Figure 1). A description for diagnosis of liver-predominant malignancy is included in Supplementary Material. Patients were deemed candidates for TARE based on recommendations conveyed by a multidisciplinary tumour board. Evaluated patients generally met the standard eligibility criteria to undergo TARE, including Eastern Cooperative Oncology Group (ECOG) grade <2, no ascites, total bilirubin <3 mg/dL, albumin >3 mg/dL, liver-dominant tumour burden, and life expectancy of at least 12 weeks.^19^ Patients were excluded if they did not meet the aforementioned eligibility criteria or when vascular anatomy was non-reproducible. Informed consent was waived for this retrospective study.

### Hybrid Angio-CT-derived and Couinaud-derived hepatic volumetry

**Figure 2.**
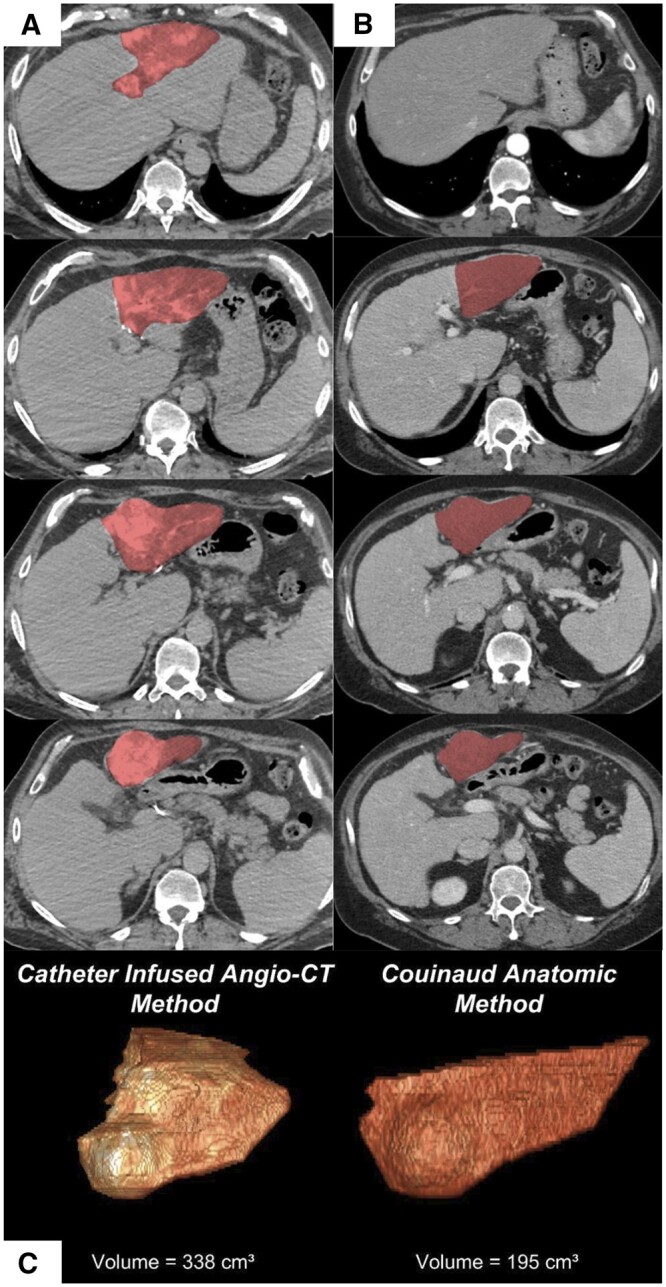
Demonstration of the differences between (A) volumetric measurement from angiosome acquired by Angio-CT vs (B) volumetric measurement derived from conventional Couinaud segment anatomy (portal venous phase; sample of contiguous cranial-to-caudal slices). (C) 3D reconstructions of the resultant delineated anatomy. In this case, the HCC tumour was visualized within segment 3; however, its tumoural angiosome is seen extending into segments 2 and 4 on Angio-CT. The unaccounted difference leads to underestimation by the Couinaud anatomic model (195 mL vs 338 mL). Note that the tumoural angiosome extends into segment 2 (red highlighted area on Angio-CT), whilst the corresponding area on diagnostic CT is not colored red for comparative purposes.

Couinaud-derived hepatic volumetry measurements were estimated based on a technique previously described by Kawasaki et al^20^ Hepatic volumes derived from Angio-CT-derived or CT/MRI-derived images were calculated using a semi-automated contouring software (Visage 7.1 Enterprise Imaging Platform, Visage Imaging, Inc., San Diego, CA). For Angio-CT-derived hepatic volumetry, contours of the perfused treatment volumes were manually delineated on every second to fourth axial CT angiographic slice. A description of the angiographic technique used with Angio-CT image acquisition is provided in Supplementary Material. For Couinaud-derived hepatic volumetry, contours of the estimated segmental or lobar treatment volumes were manually delineated on every second to fourth axial CT or MRI slice (described below). From the manually delineated contours, the software algorithm extrapolated three-dimensional geometries and volumes (cm^3^) of the regions of interest. The software-extrapolated three-dimensional contour was reviewed in axial, coronal, and sagittal reconstructions with corrections implemented as necessary. Hepatic volumes based on Angio-CT- or CT/MRI-acquired images were calculated retrospectively by three radiology resident/fellow physicians in their second, third, and fifth years of training. These volumes were reviewed independently by two board-certified diagnostic and interventional radiologists with 4 and 7 years of experience. An example highlighting the differences between the Couinaud anatomic model and perfusion model derived from Angio-CT imaging data is shown in Figure 2. Landmarks for Couinaud-derived hepatic volumetry as well as descriptions for CT and MRI imaging acquisition for treatment planning are described in Supplementary Material.

### Comparative analyses of segmental volumetry and dosimetry

Pairwise treatment-specific volume and dose differences between Couinaud and Angio-CT volumetric methods (*Vol_Couinaud_* or *Vol_Angiosome_* and *Dose_Couinaud_* or *Dose_Angiosome_*, respectively) were calculated and expressed in units of volume (mL) or dose (Gy) as follows:

$$\begin{array}{c} {\Delta \mathrm{Vol}=\;\mathrm{Vol}}_{\mathrm{Couinaud}}-{\mathrm{Vol}}_{\mathrm{Angiosome}}\\ \Delta \mathrm{Dose}={\mathrm{Dose}}_{\mathrm{Couinaud}}-{\mathrm{Dose}}_{\mathrm{Angiosome}} \end{array}$$

Differences in volume were further normalized by expressing this value as a percentage relative to the true perfusion volume to the tumour (*Vol_Angiosome_*), using the following calculation:

$$\frac{{\mathrm{Vol}}_{\mathrm{Couinaud}}-{\mathrm{Vol}}_{\mathrm{Angiosome}}}{{\mathrm{Vol}}_{\mathrm{Angio}s\mathrm{ome}}}\times 100\%$$

Differences in lobar and segmental volumes and doses derived from the Couinaud and Angio-CT-based perfusion volumes were compared.

Segmental treatment volumes and doses were categorized into subgroups based on whether the Couinaud anatomic model under- or overestimated the true volume or dose. These volume and dose differences were represented on waterfall plots. Median values of these subgroups were calculated. Dosimetry calculations are described in further detail in Supplementary Material.

### Evaluation of factors affecting differences in segmental volumetry measurement

Quantitative and qualitative analyses were employed to evaluate factors associated with segmental volumetric measurement differences. Cases demonstrating volume differences that are greater than the median overestimated or less than the median underestimated volume differences were further scrutinized (Supplementary Table S1). In addition to the independent variables discussed below, the following factors were also assessed qualitatively: tumour location (central vs peripheral), central necrosis, and central hypovascularity. Central and peripheral tumours were defined, respectively, as those either proximal or distal to the distal aspects of the right and left portal veins.

The following independent variables were assessed for correlation with volume measurement differences: tumour type (cholangiocarcinoma or HCC), near watershed location, greatest single diameter, total diameter, anatomic changes, variant vascularity, tumour multiplicity, and pretreatment imaging modality (CT vs MRI). Tumour type, pretreatment imaging modality, as well as presence or absence of anatomic changes or variant vascularity were assessed as categorical variables; the remaining independent variables were continuous values. “Near watershed” was defined as those tumours with margins (as demonstrated on pretreatment CT or MRI studies) within 1 cm of and without extension beyond a boundary between two segments within a single lobe. Anatomic liver changes included cirrhosis (*N *=* *26 segmental TARE cases), hepatomegaly secondary to a large right lobe tumour (*N *=* *2), resection of segments 5/6 (*N *=* *2), and extrinsic compression of the posterior right liver lobe due to a markedly polycystic right kidney (*N *=* *1). For segmental TARE, 21 of 56 cases were associated with variant vascular anatomy, as summarized in Supplementary Table S2.

### Statistical analysis

Differences in lobar and segmental volumes and doses derived from the Couinaud and Angio-CT-based perfusion volumes were evaluated by the two-tailed Student *t* and Wilcoxon signed-rank tests, respectively. Multiple variable linear or logistic regression was employed to assess the correlation between independent variables and differences in segmental volumetry measurements. Simple linear regression was also implemented to assess the relationship between tumour diameter and total diameter with differences in segmental volumetry measurements. A *P* value <.05 was used to indicate statistically significant differences. Statistical analyses were employed using GNU Project PSPP, version 1.4.1 (retrieved from https://www.gnu.org/software/pspp/). Graphs were generated using GraphPad Prism5 (GraphPad Software, San Diego, CA).

## Results

From September 2018 to March 2021, 72 patients underwent 110 total treatments (Figure 1). All target liver lobes and all tumours were completely included within the field-of-view by intraprocedural Angio-CT. Forty-eight patients received lobar, and 40 received segmental treatments. Five lobar and 4 segmental treatments were excluded from analysis due to non-producible vascular anatomy. The final cohort was composed of 44 patients who had undergone a total of 45 lobar treatments and 38 patients who had undergone a total of 56 segmental treatments. Patient clinical characteristics and technical details of pretreatment imaging studies are summarized in Tables 1 and 2, respectively.

**Table 1.** Patient clinical characteristics.

Clinical characteristics	Value		
**Sex**			
Male	43 (66.2%)		
Female	22 (33.8%)		
Age (years, mean, range)	65.3 [25, 84]		
**Liver malignancy**			
HCC	42 (64.6%)		
Cholangiocarcinoma	15 (23.1%)		
Colorectal	4 (6.2%)		
ACC	1 (1.5%)		
Ovarian	1 (1.5%)		
Pancreatic	1 (1.5%)		
Uveal melanoma	1 (1.5%)		
**HCC etiology**			
HCV	20 (47.6%)		
EtOH	9 (21.4%)		
NASH	8 (19.0%)		
Multiple^a^	3 (7.1%)		
Autoimmune	1 (2.4%)		
Cryptogenic	1 (2.4%)		

**Tumour size metrics** ^b^	**Median**	**Mean ± SD**	**Range**

Greatest single diameter (cm)	4.0	4.4 ± 3.5	[1.1, 19.5]
Total diameter (cm)	9.5	11.6 ± 9.6	[3.2, 53.6]
Patients with single tumour	43 (66.2%)		
Patients with multiple tumours	22 (33.8%)		

Abbreviations: HCC = hepatocellular carcinoma, ACC = adenoid cystic carcinoma of sphenoid sinus, HCV = hepatitis C virus, EtOH = alcoholic steatohepatitis, NASH, non-alcoholic steatohepatitis, SD = standard deviation.

a Three patients had one of the following: EtOH/NASH, hepatitis B virus/EtOH/NASH, HCV/NASH.

b Sizes for tumours treated by segmental TARE.

**Table 2.** Technical details of pretreatment imaging studies.

Pretreatment imaging modality for Couinaud volumetry			
**CT**			
Total	56 (54.5%)		
Segmental only	36 (64.3%)		
**MRI**			
Total	45 (44.6%)		
Segmental only	20 (35.7%)		
**Angio-CT**			
Total treatments	101		
Patients	65		
**Treatments by lobe or Couinaud segment**			
Left lobar treatments	15 (33.3%)		
Right lobar treatments	30 (66.7%)		
Segment 1	0 (0%)		
Segment 2	1 (1.8%)		
Segment 3	4 (7.1%)		
Segment 4	9 (16.1%)		
Segment 4a	2 (3.6%)		
Segment 4 b	2 (3.6%)		
Segment 5	3 (5.4%)		
Segment 6	4 (7.1%)		
Segment 7	5 (8.9%)		
Segment 8	11 (19.6%)		
Segments 2/3	1 (1.8%)		
Segments 5/6	3 (5.4%)		
Segments 5/8	7 (12.5%)		
Segments 6/7	4 (7.1%)		

**Lung shunt metrics*^a^***	**Median**	**Mean ± SD**	**Range**

Shunt fraction (%)	4.2	5.3 ± 5.5	[0.7, 30.6]
Dose (Gy)	3.5	5.1 ± 5.2	[0.4, 30.3]

a Lung shunt metrics for tumours treated by segmental TARE.

### Lobar and segmental volume and dose differences

**Figure 3.**
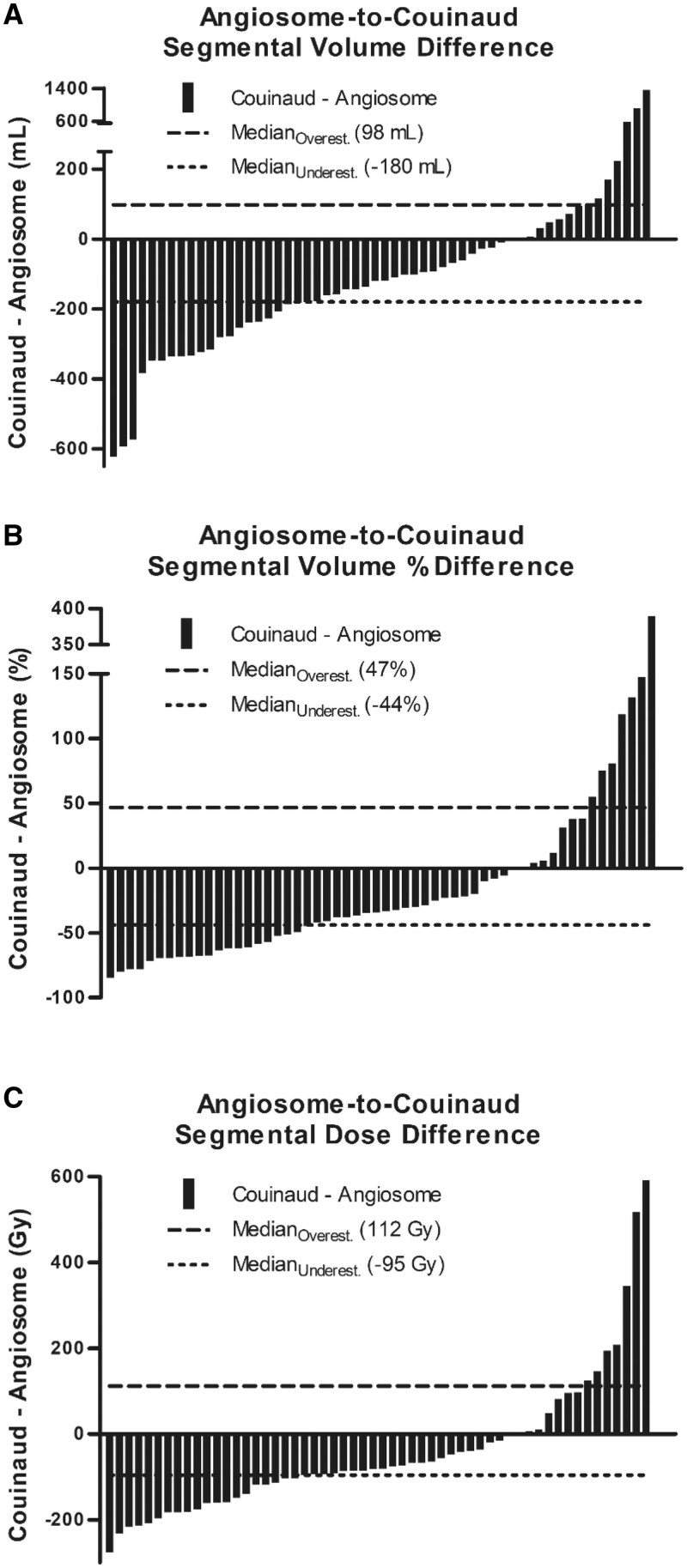
Waterfall plots of segmental treatment-specific differences by (A) volume, (B) normalized volume by percentage, and (C) dose between Couinaud-derived and Angio-CT (tumoural angiosome) volumetric measurements. Medians for the subgroups overestimated (dashed lines) and underestimated (dotted lines) by conventional Couinaud-derived volumes are provided. Forty-two volume and dose measurements were underestimated by the Couinaud anatomic model, and 14 were overestimated. Additional descriptions of outlier cases are summarized in Supplementary Table S1.

Segmental treatment-specific differences by volume, normalized volume (by percentage change in volume relative to the tumour perfusion volume derived from Angio-CT imaging), and treatment dose were analysed by Waterfall plots (Figure 3). Waterfall plots for lobar treatment-specific differences are shown in Supplementary Figure S1. Note that 62.2% (28 of 45) and 75.0% (42 of 56) lobar and segmental treatment volumes were underestimated by the Couinaud anatomic model, respectively (Table 3). Comparative analyses of lobar and segmental volumetry and dosimetry calculations are summarized in Table 4. Whilst segmental volumes and treatment doses were significantly different between the Couinaud and tumour perfusion volumetry methods (*P *<* *.0001 and *P *<* *.01, respectively), there were no differences for lobar treatments. 85.7% (48 of 56) of segmental treatment volumes and 46.7% (21 of 45) of lobar treatment volumes differed by 20% or more when compared to anatomic segmentation.

**Table 3.** Treatment-specific volume and dose Couinaud-to-angiosome error differences.

	Median Couinaud-to-angiosome over- and underestimated error differences					
	Volume (mL)		Volume percentage		Dose (Gy)	
	Over	Under	Over	Under	Over	Under
Lobar	79 [1, 671]	−214 [−786, −12]	14 [0.3, 235]	−19 [−52, −2]	22 [0, 619]	−30 [−79, −5]
Segmental	98 [4, 1 368]	−180 [−623, −1]	47 [1, 390]	−44 [−85, −0.4]	112 [4, 592]	−95 [−277, −1]

Ranges denoted in brackets.

The number of over- and underestimated lobar volumetry differences was 17 and 28, respectively. The number of over- and underestimated segmental volumetry differences was 14 and 42, respectively.

**Table 4.** Lobar and segmental volumetry and dosimetry comparative analyses.

Lobar or segmental volume	Couinaud-derived volumetry (mL)	Angiosome-derived volumetry (mL)	*P*
Right lobar (*N *=* *30)	1047 ± 415	1244 ± 449	.08
Left lobar (*N *=* *15)	571 ± 49	482 ± 40	.17
Segmental (*N *=* *56)	316 ± 313	404 ± 201	<.0001
**Lobar or segmental volume**	**Couinaud-derived dosimetry (Gy)**	**Angiosome-derived dosimetry (Gy)**	***P***
Right lobar (*N *=* *30)	148 ± 32	134 ± 73	.31
Left lobar (*N *=* *15)	197 ± 80	278 ± 247	.24
Segmental (*N *=* *56)	253 ± 74	212 ± 177	<.01

Values are expressed as mean ± standard deviation. Two-tailed Student *t* and Wilcoxon signed-rank tests were implemented for lobar and segmental comparative analyses, respectively.

### Variables affecting volume estimation error by the Couinaud anatomic model

Potential variables affecting volume estimation error by the Couinaud anatomic model were assessed by multiple variable linear regression analysis (Table 5). Tumours classified to be near watershed areas between segments were significantly correlated with underestimated volumes by the Couinaud anatomic model (*P *<* *.001). In contrast, larger tumours (by greatest diameter or total diameter) were significantly correlated with overestimated volumes (*P *<* *.0001). Near watershed tumours measured 4.1 ± 1.4 cm (range 1.8-6.8 cm) in greatest single diameter and 10.5 ± 4.3 cm (range 3.8-17.6 cm) in total diameter. There was no correlation observed for tumour type (HCC vs cholangiocarcinoma), anatomic changes, variant vascularity, tumour multiplicity, or pretreatment imaging modality.

**Table 5.** Summary of multiple variable linear regression analysis for predicting Couinaud-to-angiosome volume error.

Independent variable	*B*	95% CI	*β*	*t*	*P*
Cholangiocarcinoma	0.0	[0.0, 0.0]	−.14	−1.07	.29
HCC	0.0	[0.0, 0.0]	−.05	−0.37	.71
Near watershed	0.0	[0.0, 0.0]	−.49	−4.14	<.001
Greatest diameter	0.03	[0.02, 0.04]	.67	6.55	<.0001
Total diameter	0.08	[0.06, 0.11]	.68	6.73	<.0001
Anatomic changes	0.0	[0.0, 0.0]	.14	1.05	.30
Variant vascularity	0.0	[0.0, 0.0]	−0.24	−1.79	.08
Tumour multiplicity	0.0	[0.0, 0.0]	−.25	−1.90	.06
CT vs MRI	0.0	[0.0, 0.0]	.06	0.45	.66

Abbreviations: *B* = unstandardized coefficients, CI = confidence interval for *B*, HCC = hepatocellular carcinoma.

**Figure 4.**
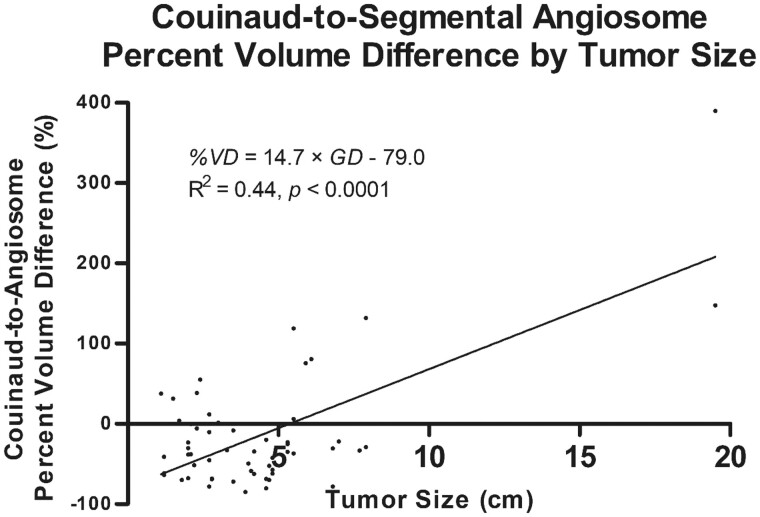
Linear regression analysis of Couinaud-to-angiosome percent volume differences by single greatest tumour dimension. *GD* represents greatest single diameter, and *%VD* represents percent volume difference.

Simple linear regression was used to assess the relationship between the greatest single diameter (*GD*) or total diameter (*TD*) of each tumour and percent volume difference (*%VD*) (Figure 4). The fitted regression models were as follows: (1) %*VD *=* *14.7 × *GD*—79.0, which was statistically significant (*R*^2^ = 0.44, *F*(1, 54) = 42.9, *P *<* *.0001), and (2) %*VD *=* *5.4 × *SD*—76.8, which was statistically significant (*R*^2^ = 0.46, *F*(1, 54) = 45.3, *P *<* *.0001). There was a significant positive correlation between greatest single diameter or total diameter and percent volume difference. Volumes of small tumours tended to be underestimated by the Couinaud anatomic method, whilst large tumours were overestimated.

**Figure 5.**
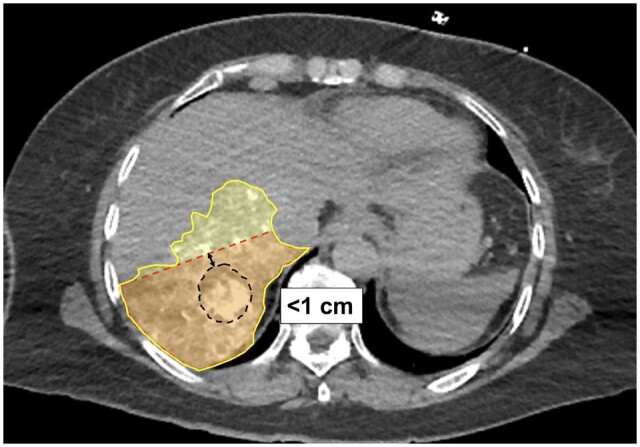
Tumours located within 1 cm of a watershed junction were significantly associated with underestimated volumetric measurements based on Couinaud anatomic method. In this case, a 3.9-cm HCC tumour (black dashed line) was seen centrally within segment 7, although it is within 1 cm of the watershed regions of segments 6/7 and 7/8 (Patient 1 from Supplementary Table S1). The tumoural angiosome acquired by pre-TARE treatment Angio-CT (yellow area) extended from segment 7 (as defined by conventional Couinaud landmarks, orange-red area) into segment 8.

Qualitative analysis of outlier cases was also implemented to scrutinize factors leading to under- or overestimation of tumour volumes by the Couinaud anatomic method. The most frequently identified feature common to tumour volumes underestimated by the Couinaud model was its location near a watershed area (52.3% or 22 of 42 underestimated volumes). Factors that led to volume overestimation by the Couinaud model included peripheral tumour location and segmental or subsegmental arterial branch selection for angiography. A representative case is shown in Figure 5: a 3.9-cm HCC tumour was seen centrally within segment 7, although it was within 1 cm of the watershed regions of segments 6/7 and 7/8. The tumoural angiosome acquired by pre-TARE treatment Angio-CT extended from segment 7 into segment 8.

**Figure 6.**
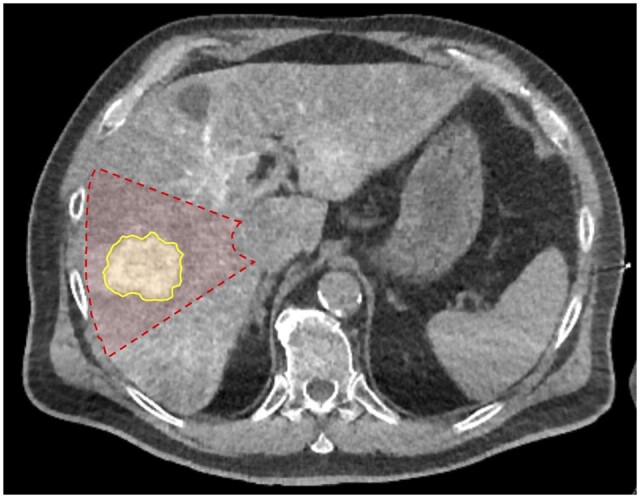
Volume measurements of peripherally located tumours were overestimated by the Couinaud anatomic model when selective angiography was performed (branch of the anterior division of the right hepatic artery in this case). Shown is a 5.5-cm HCC tumour of segments 5/8 and its corresponding tumoural angiosome, as seen on Angio-CT (yellow area; Patient 21 from Supplementary Table S1). The Couinaud segment is shown in red.

**Figure 7.**
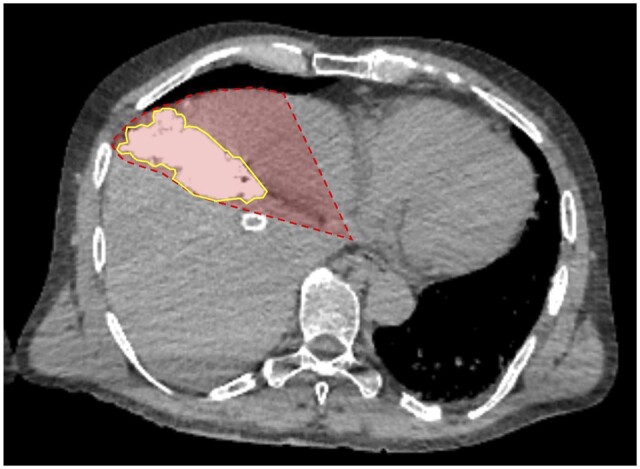
Volume measurements of peripherally located tumours were overestimated by the Couinaud anatomic model when selective angiography was performed (branch of the segment 4 arterial branch in this case). Shown is a 5.9-cm HCC tumour of segment 4, confined to its posterolateral aspect and its corresponding tumoural angiosome, as seen on Angio-CT (yellow area; Patient 19 from Supplementary Table S1). The Couinaud segment is shown in red.

Volume measurements were frequently overestimated by the Couinaud anatomic model when selective angiography (57.1% or 8 of 14 overestimated volumes) was performed for tumours located peripherally. In one case, a branch of the anterior division of the right hepatic artery was selected to infuse a 5.5-cm HCC tumour located peripherally in segments 5/8. The Couinaud anatomic model overestimated the true tumoural angiosome (Figure 6). Similarly, in another case, a branch of the segment 4 arterial branch was selected to infuse a 5.9-cm HCC tumour of segment 4. The tumoural angiosome was confined to the posterolateral aspect, leading to overestimation of the treatment volume by the Couinaud anatomic model (Figure 7).

**Figure 8.**
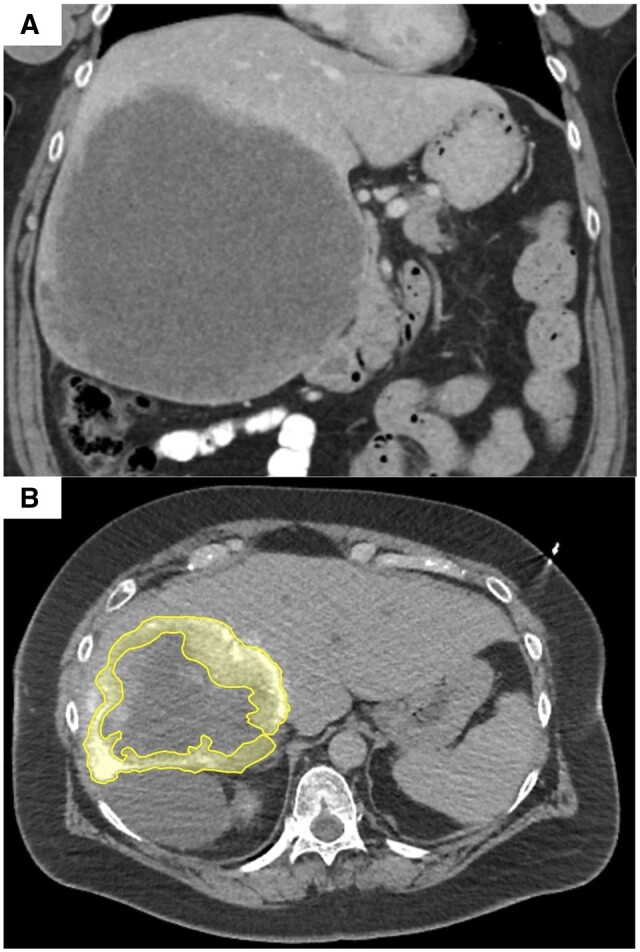
Large areas of central necrosis were major contributors to overestimated volume measurements by the Couinaud anatomic model. (A) Shown in coronal CT images acquired during portal venous phase is a 19.5-cm ovarian metastatic tumour of the entire right lobe. (B) Its corresponding tumoural angiosome, as seen on Angio-CT (yellow area; Patient 23 from Supplementary Table S1), encompasses only a minority of the true tumour volume.

**Figure 9.**
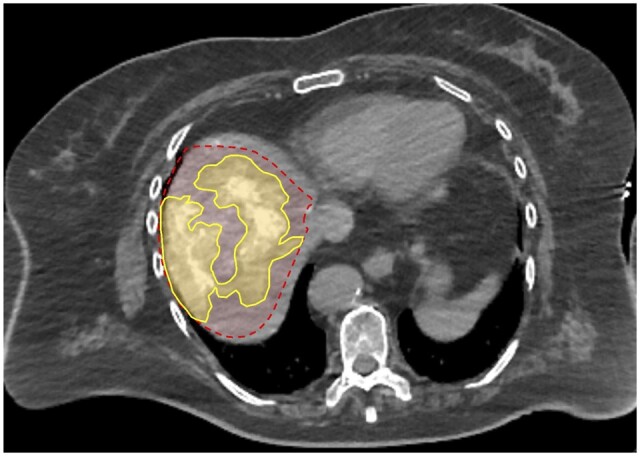
Large areas of hypovascularity within the tumour were major contributors to overestimated volume measurements by the Couinaud anatomic model. Shown is a 7.9-cm cholangiocarcinoma tumour of the dome of segment 8. Its corresponding tumoural angiosome, as seen on Angio-CT (yellow area; Patient 22 from Supplementary Table S1), encompasses only a portion of the true tumour volume. The lateral aspect of the tumoural angiosome was supplied by an arterial branch to segment 8, whilst the medial aspect was supplied by an arterial branch of segment 4. The corresponding Couinaud segment is shown in red.

Central tumour hypovascularity and necrosis were additional factors less commonly leading to volume overestimation (one instance of each). Whilst the Couinaud model accounted for the entirety of the tumour volume, including undervascularized or necrosed components, only the tumour periphery demonstrated appreciable perfusion on angiography. The impact of central tumour necrosis was evident in the case of a large 19.5-cm ovarian metastatic tumour encompassing the entire right lobe (Figure 8). In another case, a large area of central hypovascularity was observed for a 7.9-cm cholangiocarcinoma tumour of segment 8 (Figure 9). The lateral aspect of the tumoural angiosome was supplied by an arterial branch to segment 8, whilst the medial aspect was supplied by an arterial branch of segment 4.

## Discussion

There was wide variability between treatment volumes derived from the Couinaud anatomic model and those derived from Angio-CT infusion mapping (tumoural angiosome). Specifically, segmental volume measurements were significantly underestimated by the Couinaud model when compared to the tumoural angiosome (316 vs 404 mL, *P *<* *.0001), a discrepancy attributable to the greater variability of segmental volumes compared to those of lobar volumes. This is consistent with prior studies that demonstrated lobar angiosomes to vary less than segmental angiosomes relative to their Couinaud counterparts for TARE pretreatment mapping.^8^^,^^9^

There are several clinical implications of these findings. First, in practice, Angio-CT infusion mapping may replace the need for current preprocedural cross-sectional CT and MRI entirely, if this has not already been performed. However, if current preprocedural cross-sectional CT/MRI are already available, there would be partial redundancy of Angio-CT imaging in this role, as Angio-CT can provide all necessary information of traditional cross-sectional imaging. Secondly, the potential advantages of Angio-CT for personalized dosimetry may be generalizable to other SIRT devices (Y-90 resin microspheres or Ho-166 poly-L-lactic acid) provided that dosimetric calculations are derived from tumour volume.^21^ Thirdly, in light of frequent underdosing suggested by the Couinaud anatomic method, some microparticle devices may be more advantageous for treatment in cases when a high dose must be delivered to a small volume, e.g., radiation segmentectomy. Due to their higher specific activity per particle, ^90^Y glass microspheres higher doses may be achieved when compared to devices of lower activity per particle, such as Y-90 resin microspheres or Ho-166 poly-L-lactic acid.^21^ Lastly, the findings align with recent studies and guidelines, including those of the Society of Interventional Radiology as well as the Cardiovascular and Interventional Radiological Society of Europe, that have advocated for the use of cross-sectional intraprocedural imaging to define volumetry for liver-directed therapies.^17^,^22–24^

An additional ramification of these findings relates to potential misadministration of radioactive materials. The US Nuclear Regulatory Commission requires written record and prompt report of misadministration of radioactive materials, which is in part defined as a diagnostic dose of a radiopharmaceutical differing from the prescribed dose by more than 20%.^25^ Should the prescribed dose derive from the tumoural angiosome volume as opposed to anatomic segmentation, then a substantial proportion of conventionally administered TARE treatments may be retrospectively considered “misadministered” (up to 85.7% of segmental doses in the current investigation differed by at least 20% when calculated based on tumoural angiosomes).

Quantitative and qualitative analyses indicated several factors impacting differences between Couinaud-derived volumetry and the tumoural angiosome. There was a significant association of medium strength between volume differences and tumour size (%*VD *=* *14.7 × *GD—*79.0, *R*^2^ = 0.44, *P *<* *.0001). Smaller tumour volumes were likely to be underestimated by the Couinaud anatomic model, whilst larger tumour volumes were likely to be overestimated. This may in part be due to larger tumours (measuring 7.9 and 19.5 cm in greatest tumour diameter in this study) exhibiting central hypovascularity or necrosis, which was associated with volume overestimation by the Couinaud model. In these instances, the true infused volumes were confined to the tumour periphery and were substantially less than the volumes predicted by the corresponding Couinaud segments. As a result, large tumours exhibiting central hypovascularity or necrosis were more likely to be inaccurately overdosed by the Couinaud anatomic model, the consequence of which is compounded by the higher propensity for radiation-induced liver disease given the inherently diminished hepatic reserve afforded by such large tumours.^26^

Tumour location was a notable determinant of volume discrepancies. Those located near watershed areas were significantly associated with underestimated volumes by the Couinaud model both independently (*P *<* *.0001) and by multiple variable regression analysis (*β* = −.49). The majority of underestimated tumour volumes (52.3% or 22 of 42 cases) were found to be near watershed areas, emphasizing the importance of this location. A previous study described tumours within the watershed location to have underestimated tumoural angiosome volumes by the Couinaud model for TARE pretreatment imaging studies utilizing CBCT.^8^ Additionally, complete response and disease-free survival are significantly decreased for patients with watershed vs non-watershed HCC following transhepatic arterial chemoembolization.^27^^,^^28^ This is the result of arterial variations and crossover arterial supply between adjacent segments not encompassed by the Couinaud model.^29–31^ The results of the present study corroborate these findings and demonstrate a statistically significant relationship between watershed tumour location and TARE dosimetry.

Tumours located peripherally and infused via a segmental or subsegmental arterial branch led to overestimated volumes via the Couinaud model. This was a common finding with 57.1% (8 of 14) of overestimated volumes being peripheral tumours infused via selective angiography. This is concordant with a prior study, whereby the most influential factor affecting the accuracy of segmental Couinaud volume estimates was treatment peripheral to segmental artery origins for tumours at or near the liver capsule, accounting for 35.7% (10 of 28 overestimated segmental treatment volumes).^8^ These findings therefore indicate that when the Couinaud anatomic model is utilized for dosimetry, peripheral tumours accessible by superselection may be more susceptible to overdosing due to overestimated dosimetry calculations.

The increased field-of-view of Angio-CT over CBCT may be a notable advantage for TARE mapping with respect to target volume calculations.^11^^,^^12^^,^^17^ Whilst no patients in the current investigation were excluded due to inadequate coverage of the liver or tumoural angiosome, prior studies utilizing CBCT were restricted by its smaller field-of-view. Stein et al reported 26.8% of lobar and 15.4% of segmental treatment patients (11 of 41 initial lobar and 8 of 52 initial segmental angiograms excluded due to patient body habitus or inadequate coverage of the full angiosome, respectively) were excluded as a direct result of the limited field-of-view of CBCT.^8^ Additionally, one CBCT angiogram obtained for segmental treatment was excluded due to extensive lipiodol artefact, a phenomenon more pronounced with CBCT than with multidetector CT.^32^ Similarly, in a study by Ertreo et al, 7.8% of patients undergoing lobar treatment (4 patients excluded with a final cohort size of 47 patients) were excluded due to inadequate coverage of the region of interest.^9^ This represents a sizeable proportion of patients unable to undergo precise TARE dosimetry based on infusion mapping due to the limitations of CBCT. Furthermore, larger tumours were readily imaged with median and range of total diameters of 9.5 cm and 3.2-53.6 cm, respectively. In contrast, the study by Stein et al excluded larger tumours with a final cohort of substantially smaller tumours (median and range of total diameters were 5.0 cm and 2.1-7.1 cm, respectively).^8^ An important implication of this size limitation is the inability to accurately estimate infusion volumetrics of large tumours. As demonstrated in this study, volumetric estimates of larger tumours exhibiting central hypovascularity or necrosis were overestimated by the Couinaud anatomic model, a finding precluded by previous investigations due to the size limitations of CBCT. Whilst not manifested in the current or previously reported CBCT-based investigations, an additional noteworthy consideration is intraprocedural imaging invalidated by significant patient motion or respiratory artefact. This is not an uncommon occurrence for images acquired by CBCT and would have the effect of excluding additional cases from volumetric measurements derived from intraprocedural imaging.

The use of Angio-CT for TARE pretreatment measurements was also associated with more frequent and greater degrees of underestimated volumes relative to CBCT: 75% (42 of 56 segmental volumes) were underestimated by a median volume and median volume percentage of −180 mL and −44% vs 30% (12 of 40) of segment volumes underestimated by a median volume and median volume percentage of −69 mL and −20% by CBCT.^8^ The use of Angio-CT is hypothesized to have consistently delineated subtle areas of tumoural angiosome not well defined by CBCT, resulting in more frequent tumour volume underestimation by the Couinaud anatomic model. This is presumably the result of diminished contrast-to-noise ratio, less precise contrast timing, and/or artefact susceptibility (respiratory motion, beam hardening, and ring) of CBCT.^12^^,^^13^^,^^15^^,^^16^^,^^18^ Distortion of anatomy by a space-occupying tumour is also expected to have an additive effect on hepatic parenchymal volume not accounted by anatomic segmentation, likewise resulting in underestimated tumour volumes. As a result of volume underestimation, underdosing for radiation segmentectomy is a hypothesized consequence for CBCT-derived tumour volumes. The implied error introduced by the differences in dosimetry between the two modalities is on an order of magnitude that may influence TARE treatment dose-response, as demonstrated in the TARGET and DOSISPHERE-1 trials (ie, 205 Gy vs 250-300 Gy).^3^^,^^33^

There are several limitations of this investigation. First, this was a retrospective study conducted at a single tertiary medical centre with a relatively small patient size, which limits the generalizability of results. Furthermore, a retrospective investigation is predominantly restricted to only indirect comparisons between Angio-CT and CBCT. A rigorous direct comparative analysis is most achievable through a prospective study design to capture imaging data from both intraprocedural CBCT and Angio-CT for a single patient within a timeframe when the tumour is stable. In the rare case when data from both imaging modalities are available for comparison, often the timeframe between acquisition is one month or greater. Nevertheless, within the limitations of a retrospective analysis, the present study provides new information and guidance for future studies to assess these modalities. Secondly, this investigation incorporated a heterogeneous population with a spectrum of both primary and secondary hepatic malignancies. Lastly, the relatively short post-treatment follow-up period for a portion of study subjects precluded analysis of treatment outcomes and complications.

In conclusion, hepatic segmental volumes derived from the Couinaud anatomic model substantially deviated from the true tumour angiosomes delineated by Angio-CT treatment mapping. Furthermore, the increased field-of-view of Angio-CT over CBCT may be a notable advantage specifically for TARE mapping. These differences are expected to result in more accurate prescribed and delivered segmental Y-90 radioembolization doses that may ultimately lead to more optimal outcomes with respect to efficacy and safety.

## Supplementary Material

tqad056_Supplementary_Data
