# Supplementary material for: Quantitative differences in volumetric calculations for radiation dosimetry in segmental Y90 treatment planning using hybrid angiography-CT compared with anatomic segmentation
Source: Br J Radiol. 2024 Jan 4;97(1154):353–62. doi: 10.1093/bjr/tqad056 (PMC11027260; doi:10.1093/bjr/tqad056)
Supplement: tqad056_Supplementary_Data [file tqad056_supplementary_data.docx]

**Supplementary Material**

**Materials and Methods**

***Diagnosis of Liver-predominant Malignancy***

In accordance with the American Association for the Study of Liver Disease (AASLD) guidelines, hepatocellular carcinoma was diagnosed by the demonstration of a liver mass at least 1 cm in size as seen on contrast-enhanced MRI or on multi-phase contrast-enhanced CT. Other primary malignancy lesions were biopsied and diagnosis was confirmed based on morphologic and immunohistologic findings on pathologic evaluation by a board-certified pathologist. Metastatic disease was diagnosed based on chest, abdomen, and pelvis contrast-enhanced CT imaging studies, which were interpreted by board-certified diagnostic radiologists with subspecialty training in abdominal imaging.

***Hybrid Angio-CT Angiography Technique***

A single Angio-CT system (Infinix-i 4DCT, Canon Medical Systems Corporation, Otawara, Japan) was utilized for all cases included in this study. Standard angiographic technique was used to access the proper hepatic artery as previously described. [1] Progressively more selective angiograms were obtained using a microcatheter/microwire tandem, beginning with the proper hepatic artery to the right, middle, and/or left hepatic arteries, and finally, to the level of the segmental and subsegmental (or equivalent) arterial branches supplying the tumor of interest, when applicable. CT in arterial phase was obtained at each level (described below). Tumor location was confirmed by the presence of a tumoral blush. At each level, variant anatomy as well as abnormalities, including arteriovenous shunting, were noted. Lung shunt fraction (LSF) in each case was calculated from planar gamma camera examination following infusion of Technetium 99m-labeled macroaggregated albumin (99Tc-MAA) from the site of anticipated treatment.

Precise contrast administration protocols for Angio-CT image acquisition were adjusted based on operator discretion. However, in general, Angio-CT acquisitions were performed using 1:1 diluted sterile normal saline-Omnipaque^TM^ 300 (GE HealthCare, Chicago, IL, USA) with the following parameters: 1) with the catheter tip positioned at the celiac axis, 1.5-2 mL/sec injection rate for a total injected contrast volume of 15-20 mL; 2) with the catheter tip positioned at the right hepatic artery, 0.5-1 mL/sec injection rate for a total injected contrast volume of 5-10 mL; 3) for further superselection, 0.15-0.5 mL/sec for a total injected contrast volume of 1.5-5 mL. Arterial and portal venous phase images were acquired 12 and 20 sec following contrast injection, respectively.

***Landmarks for Couinaud-derived Hepatic Volumetry***

Landmarks for Couinaud-derived hepatic volumetry are described were defined as follows: The plane intersecting the middle hepatic vein and the gallbladder fossa was designated the boundary between right and left lobes. The caudate lobe (segment 1) composed of the area posterior to the main portal vein, bordered anteriorly by the plane demarcating the middle of the anterior wall of the inferior vena cava, extending posteromedially around the IVC. Segments 2 and 3 were defined as the areas situated left to the plane containing the left hepatic vein and the falciform ligament. Segments 4a and 4b were defined as the areas situated to the right of this plane. Segments 6 and 7 were defined as areas posterolateral to the plane of the right hepatic vein, and segments 5 and 8 were defined as areas anteromedial to this plane. The right and left portal veins were used as boundaries to delineate the superior from the inferior segments.

***CT and MRI Acquisition for Treatment Planning***

Pre- and multiphase post-contrast liver protocol CT and MRI scans were performed per department protocol. CT images were acquired on helical scanners with 0.625 mm slice thickness in pre-, late arterial, venous phase, and delayed post-contrast series. Axial, sagittal, and coronal reconstructions were performed in 2.5 mm slice thickness. 120 mL Iohexol (Omnipaque^TM^) 350 contrast was injected at a rate of 4 mL/sec. Display field-of-view ranged between 36 and 50 cm.

Pre- and dynamic post-contrast MR scans were acquired with 1.5 Tesla magnets with a 38 cm field-of-view and reconstructed to 3-mm thin axial slices. Weight-based gadoterate meglumine (Dotarem®, Guerbet, Villepinte, France) contrast material was administered up to a maximum of 30 mL intravenously. No enteric contrast was administered for any imaging study.

***Dosimetry Calculations***

Couinaud anatomic model-derived or Angio-CT-derived volumetric data were used to model dose calculations based on the medical internal radiation dosimetry (MIRD) formula [2]:

$$Required Activity= \frac{Target Dose \times Treatment Volume \times Density Conversion Factor}{50 \times\left( 1-Dose Vial Residual \right)\times(1-Lung Shunt Fraction)}$$

Required activity (gigabecquerel, GBq) represents the activity of Y-90 administered into the perfused liver volume. Target dose represents the absorbed dose (Gy), and in this study, this was generally prescribed to be at least 200 Gy for segmental treatments and 120-150 Gy for lobar treatments. The density conversion factor for liver tissue is 1.03 g/cm^3^. Patient-specific LSFs were used. Residual dose vial activity of 1% was assumed for all cases.

To estimate the deviation of Couinaud anatomic model-derived volumetric measurements on delivered dose, the required activity was re-calculated via the MIRD equation using the tumor perfusion volume derived from pretreatment Angio-CT imaging studies.

**Appendix**

**Tables**

**Table S1.** Clinical and Tumor Characteristics of Outlier Cases

| **Patient** | **Primary Malignancy** | **HCC Etiology*^a^*** | **Anatomy** | **Tumor Location** | **Tumor Dia. (cm)** | **Volume Diff. (mL)** | **% Vol. Diff.** | **Dose Diff. (Gy)*^b^*** |
| --- | --- | --- | --- | --- | --- | --- | --- | --- |
| 1*^d^* | HCC | NASH | Cirrhosis | Seg. 7 (NWS Seg. 6/7 and 7/8) | 3.9 | -594 | -85.0 | -232 |
| 2 | ACC | N/A | None | Seg. 3 (NWS Seg. 2/3) | 4.6 | -349 | -80.2 | -119 |
| 3 | Cholang. | N/A | None | Seg. 5 (NWS Seg. 4/5/8) | 6.8 | -207 | -78.1 | -214 |
| 4 | HCC | Multiple | Cirrhosis | Seg. 8 (NWS Seg. 5/8) | 2.7 | -574 | -78.1 | -198 |
| 5 | HCC | HCV | Cirrhosis | Seg. 5 (NWS Seg. 5/8) | 1.5 | -623 | -71.9 | -209 |
| 6 | HCC | NASH | Seg. 5/6 resection | Seg. 8 (NWS Seg. 5/8) | 1.8 | -348 | -69.6 | -184 |
| 7 | HCC | EtOH | Cirrhosis | Seg. 6 (NWS Seg. 6/7) | 4.5 | -238 | -69.6 | -176 |
| 8 | Cholang. | N/A | None | Seg. 4B (NWS Seg. 4A/4B) | 2.6 | -337 | -68.9 | -104 |
| 9 | Pancreatic | N/A | None | Seg. 3 (NWS Seg. 2/3) | 4.6 | -137 | -68.5 | -277 |
| 8 | Cholang. | N/A | None | Seg. 4B (NWS Seg. 4A/4B) | 1.8 | -333 | -68.1 | -103 |
| 10 | Cholang. | N/A | None | Seg. 7 | 2 | -281 | -67.7 | -83 |
| 1 | HCC | NASH | Cirrhosis | Seg. 8 (subcapsular) | 1.1 | -336 | -63.6 | -182 |
| 10 | Cholang. | N/A | None | Seg. 4 | 3.9 | -121 | -62.1 | -161 |
| 11 | Cholang. | N/A | None | Seg. 8 (NWS Seg. 7/8) | 4.8 | -279 | -62.0 | -161 |
| 1 | HCC | NASH | Cirrhosis | Seg. 8 (subcapsular) | 1.1 | -187 | -61.3 | -150 |
| 12 | HCC | Multiple | Cirrhosis | Seg. 2 (NWS Seg. 2/3) | 4.1 | -228 | -58.5 | -184 |
| 13 | Colorectal | N/A | None | Seg. 2/3 (NWS Seg. 2/3/4) | 3.8 | -317 | -57.1 | -86 |
| 14 | Cholang. | N/A | Cirrhosis | Seg. 6 (NWS Seg. 6/7) | 4.1 | -183 | -52.3 | -217 |
| 5 | HCC | HCV | Cirrhosis | Seg. 7 (NWS Seg. 7/8) | 1 | -178 | -51.4 | -159 |
| 15 | HCC | HCV | Cirrhosis | Seg. 6 (subcapsular) | 4 | -103 | -49.5 | -140 |
| 16 | Cholang. |  | None | Porta hepatis | 2.7 | -384 | -45.2 | -114 |
| 17 | HCC | HCV | Cirrhosis | Seg. 8 (subcapsular) | 2.3 | 57 | 38.5 | 98 |
| 18 | HCC | EtOH | Cirrhosis | Seg. 4B | 2.4 | 116 | 55.2 | 147 |
| 19*^d^* | HCC | HCV | Cirrhosis | Seg. 4 (confined to posterolateral aspect) | 5.9 | 171 | 75.7 | 194 |
| 20 | HCC | EtOH | Cirrhosis | Seg. 4A (subcapsular) | 6.1 | 101 | 80.8 | 208 |
| 21*^d^* | HCC | NASH | None | Seg. 5/8 (peripheral) | 5.5 | 595 | 118.8 | 519 |
| 22*^d^* | Cholang. | N/A | None | Seg. 8 (subcapsular); central hypovascularity | 7.9 | 224 | 131.8 | 345 |
| 23*^d^* | Ovarian | N/A | Hepatomegaly*^c^* | Right lobe; central necrosis*^c^* | 19.5 | 923 | 147.7 | 126 |
| 23*^d^* | Ovarian | N/A | Hepatomegaly*^c^* | Right lobe; central necrosis*^c^* | 19.5 | 1368 | 389.7 | 592 |

HCC = hepatocellular carcinoma, ACC = adenoid cystic carcinoma of sphenoid sinus, Cholang. = cholangiocarcinoma, Seg. = segment, NWS = near watershed, Dia. = greatest single diameter, Diff. = difference.

***^a^***Etiologies for HCC in patients 4 and 12 were HCV/NASH and HBV/EtOH/NASH, respectively.

***^b^***Dose difference represents the dose calculated from Angio-CT volumetry minus the dose calculated from Couinaud volumetry measurements.

***^c^***Hepatomegaly and central necrosis as a result of large right lobe metastasis.

***^d^***Additional details for patients 1, 19, 21-23 are provided in **Figures 5, S3, S2, S5,** and **S4**, respectively.

**Table S2.** Summary of Variant Vascular Anatomy in this Study

| **Variant Vascular Anatomy** | **Number of Cases** |
| --- | --- |
| Replaced RHA originating from SMA | 5 |
| Abnormal vascularity seen throughout liver secondary to cholangiocarcinoma and prior post-treatment changes | 4 |
| Replaced LHA originating from LGA | 4 |
| Accessory RHA originating from SMA | 2 |
| CHA originating from aorta; GDA originating from CHA; accessory RHA originating from celiac trunk | 2 |
| Accessory LHA originating from LGA | 1 |
| Common origin of celiac artery and SMA | 1 |
| MHA originating near origin of GDA; replaced LHA originating from LGA | 1 |
| Omental arterial branch from distal GDA supplying segment 7/8 (not tumor supplying) | 1 |

CHA = common hepatic artery, RHA = right hepatic artery, MHA = middle hepatic artery, LHA = left hepatic artery, SMA = superior mesenteric artery, LGA = left gastric artery, GDA = gastroduodenal artery

**Figures**

**(a)
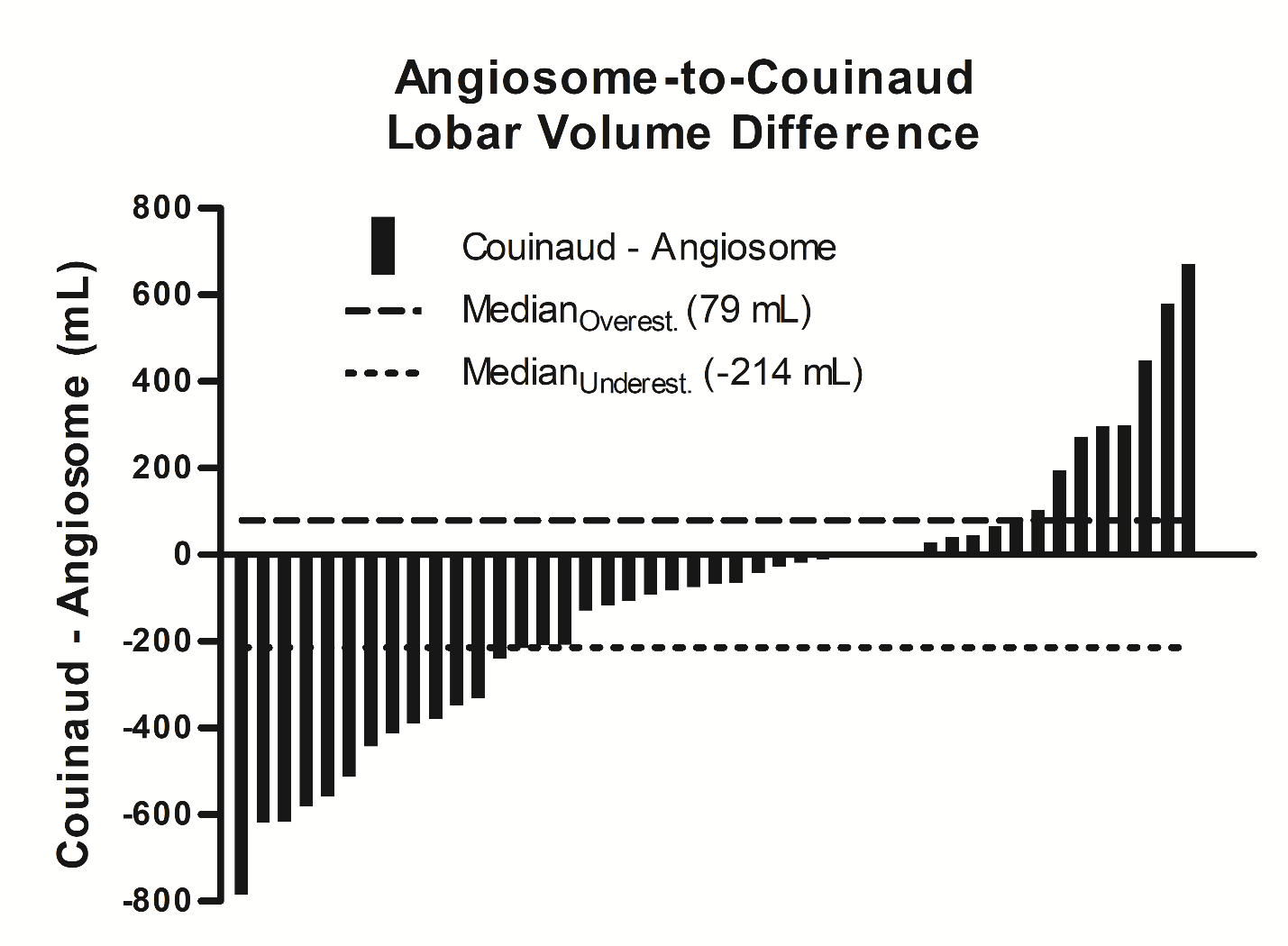
**

**(b)
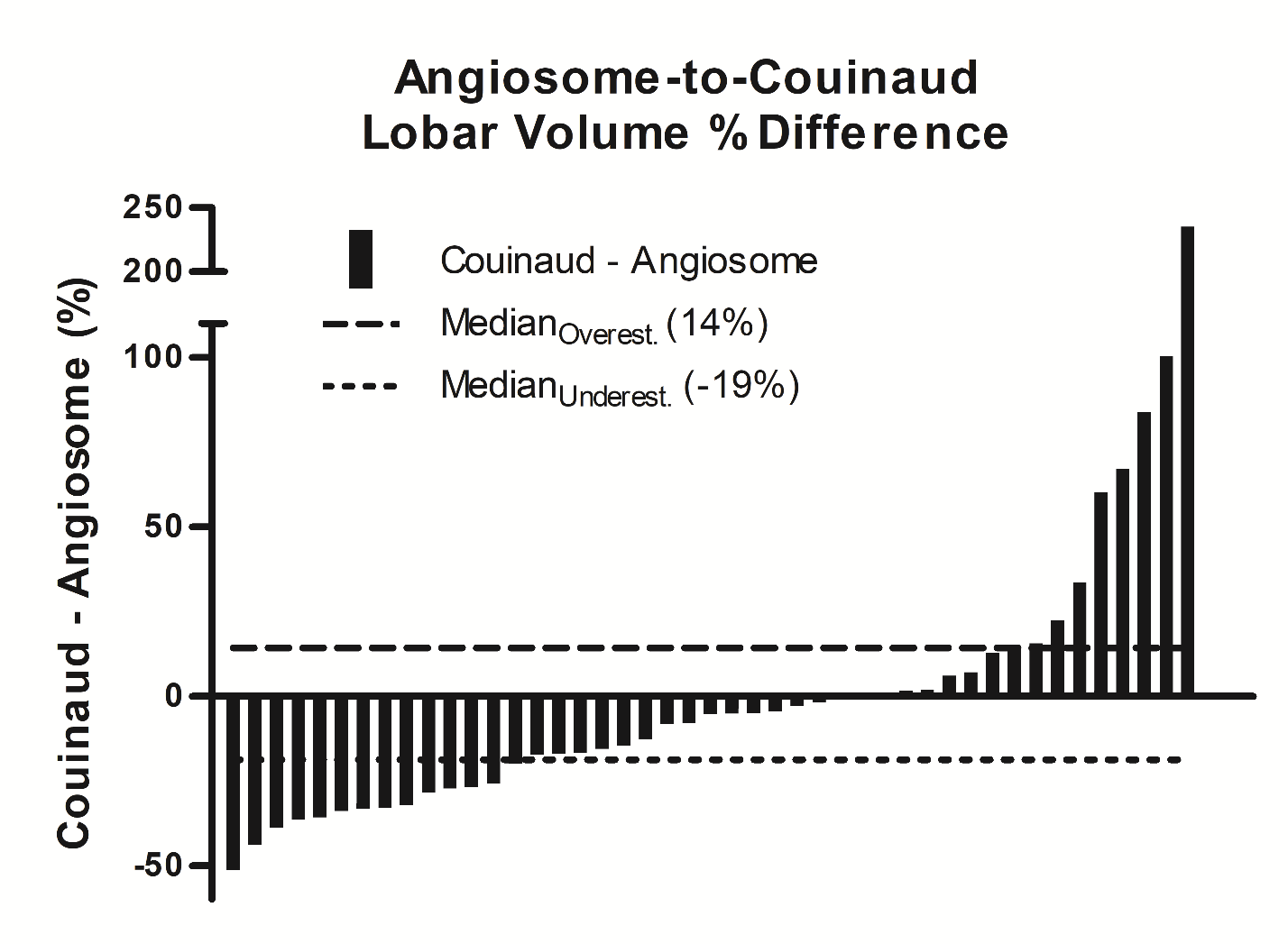
**

**(c)**

**
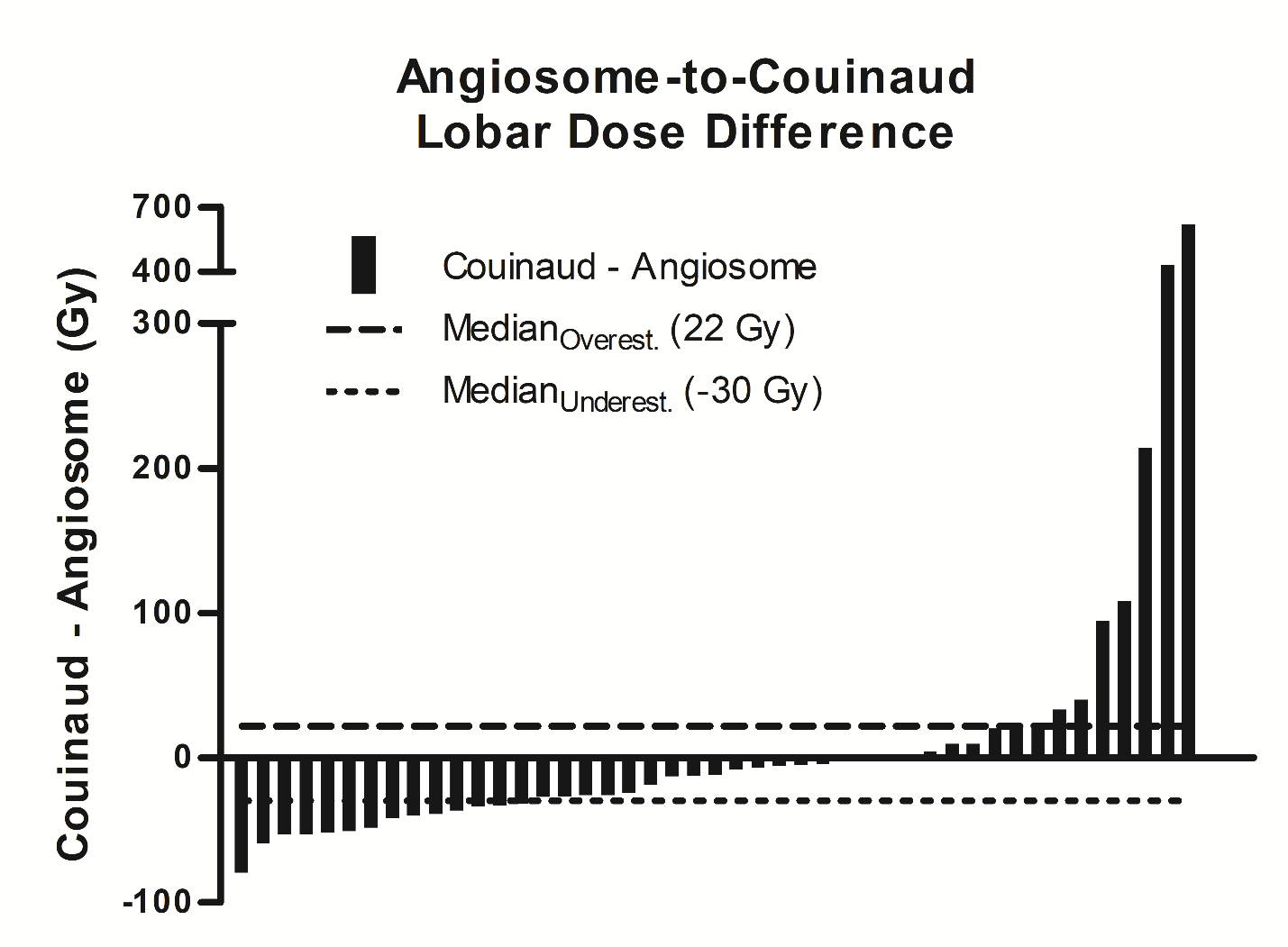
**

**Figure S1.** Waterfall plots of lobar treatment-specific differences by **(a)** volume, **(b)** normalized volume by percentage, and **(c)** dose between Couinaud-derived and Angio-CT (tumoral angiosome) volumetric measurements. Medians for the subgroups overestimated (dashed lines) and underestimated (dotted lines) by conventional Couinaud-derived volumes are provided. Twenty-eight volume and dose measurements were underestimated by the Couinaud anatomic model and 17 were overestimated.

**References**

1. Toskich BB, Liu DM. Y90 radioembolization dosimetry: concepts for the interventional radiologist. *Techniques in Vascular and Interventional Radiology.* 2019;22(2):100-111.

2. TheraSphere Interactive Dose Ordering Calculator. Boston Scientific Corporation. <https://www.bostonscientific.com/en-US/products/cancer-therapies/therasphere-y90-glass-microspheres/ordering-information/therasphere-idoc.html>. Accessed July 23rd, 2022.
